# Respiratory Epithelial Adenomatoid Hamartoma: An Uncommon Differential of Nasopharyngeal Tumor

**DOI:** 10.1155/2023/9942293

**Published:** 2023-11-30

**Authors:** Taiwo Olufemi Solaja, Kenechukwu Chinemelum Uche-Okonkwo, Moses Ayodele Akinola, John Ifeanyi Nwadiokwu

**Affiliations:** ^1^Department of Anatomic Pathology, Ben Carson College of Health and Medical Sciences, Babcock University, Ilishan-Remo, Ogun State, Nigeria; ^2^Otorhinolaryngology Unit, Department of Surgery, Ben Carson College of Health and Medical Sciences, Babcock University, Ilishan-Remo, Ogun State, Nigeria

## Abstract

Respiratory epithelial adenomatoid hamartoma (REAH) is an uncommon tumor-like lesion that has been reported within the nose, paranasal sinuses, and less frequently in the nasopharynx. While it is believed to be benign, its clinical presentation, radiological characteristics, and microscopic features may closely mimic more aggressive tumors of the upper respiratory tract, potentially leading to needless life-altering treatment. Prior to now, there had been no reported cases of this lesion in West Africa. We present a 35-year-old male with persistent bilateral nasal obstruction, difficulty with swallowing, and bilateral reduction in hearing, whose CT findings were highly suggestive of a nasopharyngeal tumor, but upon biopsy and histology showed features most consistent with REAH. Surgery completely alleviated his symptoms with no clinical evidence of recurrence after a 1-year follow-up period.

## 1. Introduction

A hamartoma is a self-limited, tumor-like malformation made up of an abnormal mixture of mature cells and tissues found in the area of the body where growth occurs [1].

Respiratory epithelial adenomatoid hamartoma (REAH) has been reported to arise from the nose, paranasal sinuses, and less commonly the nasopharynx. Even though it generally runs a benign clinical course, it shares radiological and histopathological features with a number of more biologically aggressive tumors of the upper airway. Careful and comprehensive evaluation of these patients and the specimen is necessary to avoid unwarranted life-altering treatment and morbidity.

## 2. Case Report

A 35-year-old male, known to be asthmatic, presented to our outpatient clinic on account of noisy breathing of 4 years duration which had progressed over the previous few weeks with worsening difficulty with breathing, bilateral aural fullness, difficulty with swallowing, and a muffled voice. There was also associated recurrent nasal discharge and postnasal drip.

Examination revealed dullness of both tympanic membranes and no demonstrable nasal patency. A mass was seen protruding from the nasopharyngeal isthmus, jutting into the oropharynx and tenting the soft palate.

There were no palpable neck nodes.

**Figure 1:**
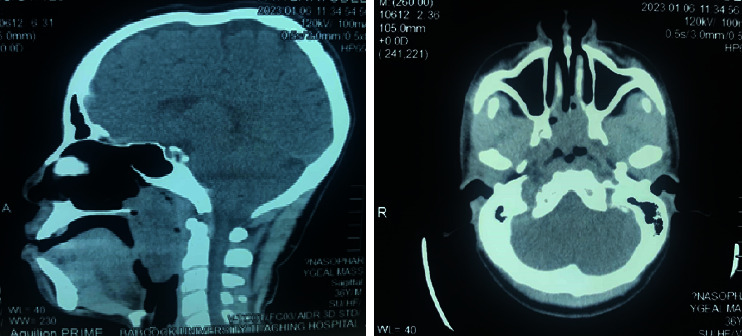
Computed tomography scan images showing a nonenhancing isodense mass lesion occupying the posterior nasal cavity, nasopharynx, and inferiorly to the oropharynx.

Contrast CT revealed a globular, nonenhancing, soft tissue density mass occupying the nasopharynx and the posterior nasal cavity bilaterally, obliterating the pharyngeal recesses and extending inferiorly to the oropharynx, displacing the soft palate anteriorly with severe pharyngeal-airway narrowing. It measured 73.8 × 26.3 × 28.6 mm (Figure 1). There were no radiologically significant cervical nodes.

**Figure 2:**
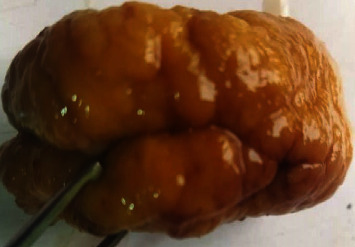
Excised “cerebriform” mass.

The patient had examination under general anesthesia and endoscopic-assisted excision of the mass, which was easily dissected whole from the adjoining mucosa and structures (Figure 2) and was sent for histopathology. He was discharged on the 1^st^ day post-op.

**Figure 3:**
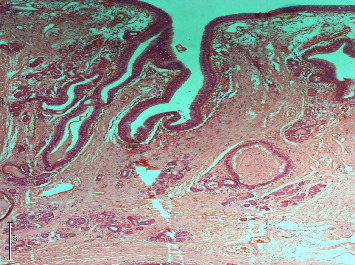
Micrograph H&E @ ×100 showing a section of the nasopharyngeal mass lined by the respiratory-type epithelium with invagination into the stroma, forming glands that have ciliated epithelial lining and outer prominent basement membrane consistent with a respiratory epithelial adenomatoid hamartoma.

Microscopy showed a loose fibrocollagenous tissue partly covered by the respiratory epithelium. The epithelium was thrown into papillomatous folds with the fibrovascular core. These folds were forming glands deep into the stroma. There were also numerous dilated glands surrounded by thick basement membranes with congested vessels and areas of hyaline change, as well as deep infiltration of the tissue with numerous lymphocytes (Figure 3).

The patient reported complete resolution of symptoms within a week postoperatively, with no features of recurrence on the follow-up for one year.

## 3. Discussion

Two major types of hamartomas have been described in the upper respiratory tract. The more common nasal chondromesenchymal hamartoma comprises primarily of cells of mesodermal origin [2]. The other respiratory epithelial adenomatoid hamartoma was first described by Wenig et al. in 1995 [3]. A year later, however, the same authors designated a subtype of REAH with admixed chondro-osseous elements chondro-osseous respiratory epithelial adenomatoid hamartoma (COREAH) [4].

While REAH is considered a rare entity, a recent rise in case reports has led to the reflection that it might be grossly underreported due to its histologic similarities with allergic nasal polyps, inverted papilloma, and sinonasal adenocarcinoma [5]. Nevertheless, as of 2023, ours is the first reported case we could find from West Africa.

The clinical features of REAH are nonspecific. Our patient presented with symptoms of progressive upper airway obstruction, aural fullness, and dysphagia. These symptoms, coupled with the clinical findings, were suggestive of a tumor arising from the nasopharynx which was confirmed by the CT scan. However, the fact that it neither showed radiologic features of infiltration nor neck node involvement indicated its benign nature. Another peculiar radiologic feature of REAH is its ability to significantly widen the olfactory clefts [6]. This has, however, been only applicable to nasal cavity and ethmoidal REAHs. A nasopharyngeal REAH so extensive that it manifests this finding has not been reported.

The gross appearance of this lesion appears to vary widely, from polypoid to nodular and cerebriform [6, 7]. The tumor excised from our patient had a characteristic cerebriform appearance of firm consistency. REAH has also been reported to coexist with inflammatory polyps, as well as with inverted papilloma, which lends further uncertainty to its possible aetiology as either a reactive inflammatory or an entirely neoplastic disease [8]. While an incisional biopsy may give a preliminary indication as to the nature of the mass (whether it is of malignant or benign aetiology), in practice, it is unlikely to preclude performing a definitive excision. In two reported cases where an incisional biopsy was performed for REAH, the preliminary histopathologic diagnoses suggested inflammatory nasal polyps and inverted papilloma, both of which were at variance with the final histologic diagnosis of REAH after complete excision [9, 10].

Definitive diagnosis of REAH is made by histopathology. Wenig et al. described these lesions as being dominated by the presence of glandular proliferation, some of which were widely dilated, all lined by ciliated respiratory epithelium and in continuity with the surface epithelium. This surface epithelium appeared to invaginate downward into the mucosa. Other features include polypoid growth, stromal edema, seromucous gland proliferation, vascular and fibroblastic proliferation, and mixed chronic inflammatory cell infiltrates [3].

Our specimen showed histologic features identical to those described by Wenig and Heffner. The challenge in making the diagnosis, however, usually lies in distinguishing it from close histologic differentials.

Inflammatory polyps and REAH both show fibroblastic and vascular proliferation, stromal edema, and infiltration with mixed inflammatory cells. Inflammatory polyps, however, do not show the florid adenomatoid proliferation and stromal hyalinization characteristic of REAH [11].

Another close differential is inverted papilloma. Both demonstrate invagination of the surface epithelium. However, inverted papilloma usually consists of squamous epithelial cells (as with the respiratory epithelium seen in REAH), interspersed with mucin-containing cells and microcysts. There is also usually some degree of atypia [11].

The role of immunochemistry is inconclusive at this time. One study evaluating the immunohistochemical and genetic profiles of 10 cases of REAH and comparing them with 9 cases of low grade tubulopapillary adenocarcinoma found evidence of clonality in 1 case, suggesting a precursor relationship between REAH and this subtype of sinonasal adenocarcinoma [12]. This provisional association requires further research.

## 4. Conclusion

It is imperative that otorhinolaryngologists and pathologists are aware of the clinical and histologic features of this rare differential when evaluating patients with symptoms suggestive of nasopharyngeal tumor.
